# 1419. High Prevalence of Fluoroquinolone-Resistant Urinary Tract Infection Among US Emergency Department Patients Diagnosed with UTI, 2018-2020

**DOI:** 10.1093/ofid/ofab466.1611

**Published:** 2021-12-04

**Authors:** Brett Faine, Megan A Rech, Priyanka Vakkalanka, David A Talan

**Affiliations:** 1 University of Iowa, Iowa City, Iowa; 2 Loyola University Medical Center, Maywood, Illinois; 3 Olive View-UCLA Medical Center, Sylmar, CA

## Abstract

**Background:**

Uropathogen resistance, Fluoroquinolone-resistance (FQR) and Extended spectrum beta-lactamase (ESBL), has been observed to be emerging worldwide with prevalences above recommended thresholds for routine empirical treatment. We sought to determine recent resistance prevalence from a geographically diverse sample of US Emergency Departments (ED).

**Methods:**

We conducted a multi-center, observational cohort study utilizing a network of 15 geographically diverse US EDs. Patients ≥ 18 years of age with the primary international classification of diseases (ICD-10) diagnosis code of cystitis, pyelonephritis, or urinary tract infection (UTI) and were discharged home from the ED from 2018-2020 were included. We calculated descriptive statistics for uropathogens and susceptibilities. Logistic regression analysis was used to identify antimicrobial resistance risk factors associated with fluoroquinolone (FQ)-resistant *Escherichia coli*.

**Results:**

Among 3,779 patients who met inclusion criteria, median age was 62.9 years (IQR: 41-77.6) and 76.3% were female. The most common diagnoses were complicated (40.9%) and uncomplicated cystitis (39.4%). Six hundred and forty-five (17%) patients reported receiving antimicrobials in the previous 90-days. *E. coli* was the most common pathogen (62.9%), followed by *Klebsiella pneumoniae* (13%) and *Enterococcus* species (5.8%). Across all sites, overall *E. coli* FQ-resistance prevalence was 22.1%, ranging from 10.5 to 29.7% by site. The prevalence of ESBL-producing uropathogen was 4.4%, ranging from 2.3% to 8.6% by site. Previous IV or oral antimicrobial use in the last 90-days and complicated vs. uncomplicated UTI were associated with FQ-resistant *E. coli* (OR 1.69, 95% CI: 1.33-2.14, and OR 1.60, 95% CI: 1.26-2.02, respectively). Of the most prescribed oral antibiotics upon patients discharged from the ED, *E. coli* resistance to nitrofurantoin and cephalexin was 1.8% and 0.9%, respectively.

**Conclusion:**

FQ-resistant *E. coli* is widely prevalent and ESBL-mediated resistance appears to be emerging across US sites highlighting the need for ongoing monitoring of antimicrobial resistance and, at some locations, modification of empirical treatments.

**Disclosures:**

**Brett Faine, PharmD**, **Spero Therapeutics** (Research Grant or Support) **Megan A. Rech, PharmD, MS, BCCCP, FCCM**, **Spero** (Research Grant or Support) **David A. Talan, MD**, **AbbVie** (Consultant)**GSK** (Consultant)**SPERO Therapeutics** (Grant/Research Support)

